# Growing public interest and regional disparities of cardiorenal syndrome in the United States

**DOI:** 10.3389/fdgth.2026.1803510

**Published:** 2026-09-15

**Authors:** Reid P. Schlesinger, Kianoush B. Kashani, Charat Thongprayoon, Wisit Cheungpasitporn, Jing Miao

**Affiliations:** 1 Department of Medicine, Mayo Clinic, Rochester, MN, United States; 2 Division of Nephrology and Hypertension, Department of Medicine, Mayo Clinic, Rochester, MN, United States; 3 Division of Pulmonary and Critical Care Medicine, Department of Medicine, Mayo Clinic, Rochester, MN, United States

**Keywords:** cardiorenal syndrome, digital epidemiology, health disparities, mortality, public awareness

## Abstract

**Background:**

Public awareness and regional engagement with cardiorenal syndrome (CRS) remain poorly understood. We examined temporal trends and geographic patterns of U.S. public interest in CRS and their relationship with disease burden.

**Methods:**

Google Trends™ data (2004–2025) annual relative search interest (RSI) for CRS. CRS-related mortality was derived from CDC WONDER (2007–2020) using hypertensive heart and chronic kidney disease deaths. Temporal trends were assessed via augmented Dickey-Fuller tests and linear regression. Associations between RSI and mortality were evaluated using Pearson correlation, AR(1) modeling, first-difference, and cumulative change analysis. State-level correlations were examined with and without adjustment for income, education, and insurance coverage.

**Results:**

Both RSI and mortality exhibited strong non-stationary upward trend (*p* = 0.98 and 0.42). Raw data showed a strong correlation analysis (r = 0.79, *p* = 0.001); but first-differenced year-over-year changes were not significant (r = −0.36, *p* = 0.23), consistent with AR(1) modeling (*β*=1.58, *p* = 0.11). Cumulative changes remained strongly correlated (r = 0.80, *p* = 0.001), indicating parallel long-term trajectories. Findings were consistent with varying ICD-10 code definitions for CRS-related mortality. Geographically, RSI was highest in West Virginia and lowest in Texas and Georgia. State-level RSI showed no correlation with age-adjusted mortality, both unadjusted (r = −0.09, *p* = 0.52) and after adjustment for median income, educational, and insurance coverage (*β*=0.03, *p* = 0.75).

**Conclusion:**

Public interest in CRS has grown in parallel with mortality, reflecting its increasing epidemiological relevance. However, the lack of geographic correlation between search interest and mortality—even after socioeconomic adjustment—suggests awareness gaps and highlights opportunities for targeted education and resource allocation.

## 1 Introduction

For Cardiorenal syndrome (CRS) describes a spectrum of disorders in which acute or chronic dysfunction of either the heart or kidneys induces dysfunction in the other organ. This bidirectional pathology, mediated by complex hemodynamic, inflammatory, metabolic, and neurohormonal mechanisms (1–3), is a major driver of increased morbidity, mortality, and healthcare costs (4–7). Despite ongoing advances in clinical management (5, 8, 9), the prevalence of CRS continues to grow, driven by the rising global burden of heart failure and chronic kidney disease (CKD) (6, 7, 10, 11). In the United States alone, acute CRS complicates 25% to 50% of the over one million annual hospitalizations for decompensated heart failure (6), while CKD underlies 45%–63% of heart failure cases (11).

Effective CRS management extends beyond medical intervention to hinge on early detection, prevention, and patient engagement—goals critically dependent on public awareness. Awareness influences symptom recognition, health-seeking behaviors, and treatment adherence, yet the extent and evolution of public knowledge about CRS remain poorly characterized. Traditional epidemiological surveillance, while essential, often lacks the timeliness to capture dynamic shifts in public attention and information-seeking behavior.

Infodemiology, the study of health-related information distribution and seeking on the internet (12–15), provides a unique, real-time lens through which to examine public engagement. Tools like Google Trends™, which aggregates anonymized search query data, provide a powerful means to quantify population-level interest in health topics. This methodology has been successfully applied to track public engagement with various kidney diseases, including kidney stones, acute kidney injury (AKI), and dialysis modalities (16–19). Such insights have the potential to guide targeted educational initiatives, inform public health strategies, and even predict changes in health trends (14).

To address the gap in understanding public awareness of CRS, this study employs Google Trends™ to analyze two decades of search data for “Cardiorenal syndrome.” Our objectives are threefold: 1) to quantify the temporal trajectory of public interest in CRS, 2) to examine the correlation between online search activity and CRS-related mortality, and 3) to map geographical disparities in search interest across the United States. The findings aim to identify regions where targeted educational interventions may be most impactful, thereby informing more effective public health strategies.

## 2 Methods

### 2.1 Ethics

This study did not include any research involving human participants or animals, so no ethical approval was required.

### 2.2 Public interest data retrieval

Public interest in CRS was quantified using Google Trends™ (20), analyzing search data for the term “Cardiorenal syndrome” from January 1, 2004, to December 31, 2025. Data were available under three classifications: Search term, Topic, and Medical Disorder. Preliminary analysis revealed minimal activity under the Topic classification and high concordance between the Search term and Medical Disorder categories (Supplementary Figure S1). Consequently, data from the Medical Disorder category were selected as the primary dataset for all subsequent analyses to ensure consistency and relevance.

The key metric was the monthly Relative Search Interest (RSI) index, a normalized measure representing search volume relative to the highest observed search frequency. RSI values range from 0 to 100, where 100 denotes peak popularity within the selected region and timeframe, 50 indicates half the peak popularity, and 0 signifies insufficient data for analysis. To analyze year-over-year trends, annual RSI values were calculated by averaging the corresponding monthly indices.

To provide international context, RSI data were also extracted for four other predominantly English-speaking countries: the United Kingdom, Canada, Australia, and New Zealand. For domestic geographic analysis, subregional RSI values within the United States were examined, similarly scaled from 0 to 100 relative to the highest-searching region.

This study utilized only aggregated, anonymized public data; thus, institutional review board approval was not required.

### 2.3 CRS-related mortality data retrieval

Data on mortality were obtained from the U.S. Centers for Disease Control and Prevention (CDC) Wide-ranging Online Data for Epidemiologic Research (WONDER) database, using the Final Multiple Cause of Death dataset (21). This user-friendly online system provides free access to CDC's extensive public health data. For this analysis, we utilized the Final Multiple Cause of Death Data, which was available from 1999 to 2020 at the time of retrieval.

CRS-related mortality was identified using the International Classification of Diseases, Tenth Revision (ICD-10) code I13.x (hypertensive heart and CKD with heart failure) as the underlying cause of death in the primary analysis. Given the absence of a specific ICD-10 code for CRS-related mortality, this definition may not capture the exact or complete clinical spectrum of the syndrome, which encompasses Type 1 (acute decompensated heart failure with AKI), Type 2 (chronic heart failure with progressive CKD), Type 3 (AKI causing cardiac dysfunction), Type 4 (CKD with chronic cardiac dysfunction), and Type 5 (secondary CRS, e.g., systemic inflammatory disease causing both heart and kidney dysfunction). To mitigate this limitation, we conducted sensitivity analyses using multiple ICD-10 definitions that included codes for heart failure (I50.x), CKD (N18.x, N19.x), and AKI (N17.x), analyzing two logical conditions: “AND” (indicted by &) and “OR” (indicted by/).

We extracted national and state-level statistics, including total death counts, corresponding population estimates, and calculated crude mortality rates and age-adjusted mortality rates per million population.

### 2.4 Definitions

*Δ*RSI was defined as the year-over-year change (change from previous year) in RSI. This first-difference transformation was applied to remove the underlying secular trend in the RSI time series and to isolate short-term fluctuations in public search interest. Positive values of *Δ*RSI indicate that search interest increased compared to the previous year, while negative values indicate a decrease. This approach addresses the non-stationarity of the raw RSI series and avoids spurious correlations driven by shared temporal trends.

Cumulative *Δ*RSI represents the net cumulative change in RSI from the 2007 baseline to each subsequent year, which is calculated by summing all year-over-year changes (*Δ*RSI) up to a given point in time.

Similarly, *Δ*Mortality was calculated to assess the year-over-year change in mortality rate, and cumulative *Δ*Mortality was defined as the net cumulative change in mortality from the 2007 baseline.

### 2.5 Statistical analysis

The temporal properties of the search interest time series were evaluated using the Augmented Dickey-Fuller (ADF) test to assess stationarity. Trends over time in both RSI and mortality rates were examined using linear regression analysis. Pearson's correlation coefficient was computed to quantify the association between the RSI values and the corresponding mortality rates. To address the potential for spurious correlation due to temporal autocorrelation, we performed a regression analysis with autoregressive AR(1) errors. The model regressed annual RSI on annual mortality while explicitly modeling the error structure as a first-order autoregressive process. Estimation was performed using maximum likelihood. The Durbin-Watson statistic was used to assess residual autocorrelation before and after modeling. State-level correlations were examined with and without adjustment for family median income, education level, and insurance coverage using ordinary least-squares linear regression; these data were extracted from the U.S. Census Bureau (https://data.census.gov/table). Statistical analyses were conducted using JMP software (SAS Institute Inc.), with a statistical significance set at *p* ≤ 0.05**.**

## 3 Results

### 3.1 Public interest in cardiorenal syndrome

Public interest in CRS over the past two decades was assessed by analyzing monthly RSI indices from five predominantly English-speaking countries: the United States, the United Kingdom, Canada, Australia, and New Zealand (Supplementary Figure S2, Table S1). While initial search activity was recorded beginning in 2015, 2018, and 2019 for the UK, Canada, and Australia respectively, the United States consistently demonstrated significantly higher search volume than the other four nations from 2007 (Figure 1a, Supplementary Table S2). Given this substantial disparity, subsequent detailed trend analyses were focused on the U.S. dataset from 2007 to 2025.

**Figure 1:**
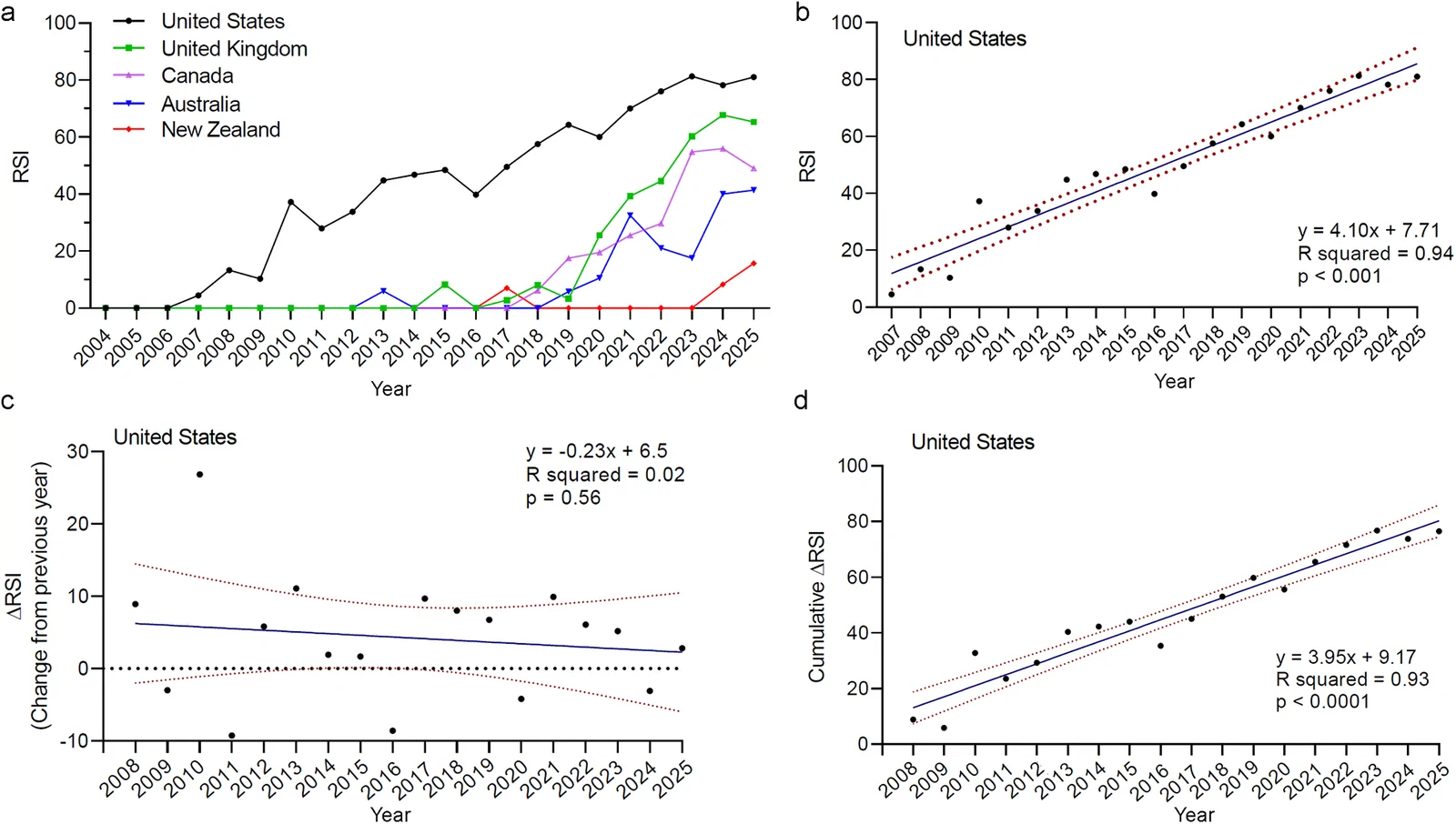
Trends in search activity for cardiorenal syndrome. **(a)** Trends in relative search interest (RSI) from Google Trends (2004–2025) in the five countries where English is the primary spoken language. **(b)** Linear regression analysis illustrating the temporal increase in search activity. **(c)** Linear regression of ΔRSI (change from previous year–2007) over time. **(d)** Linear regression of cumulative ΔRSI (net cumulative change in RSI from a baseline year to each subsequent year) showing a substantially positive trend.

To characterize the temporal properties of the search data, an AD*F* test was performed. The test yielded a *p*-value of 0.98, with an ADF statistic of 0.35, exceeding the critical thresholds at the 1%, 5%, and 10% significance levels (−4.33, −3.23 and −2.75, respectively). This confirms the non-stationarity of the time series, indicating the presence of an underlying trend. Subsequent linear regression analysis revealed a statistically significant and visibly positive upward trajectory in RSI over time (slope = 4.10, R^2^ = 0.94, *p* < 0.001), supporting the observation of increasing public interest (Figure 1b).

Analysis of the year-over-year changes in RSI (*Δ*RSI) revealed that search interest increased in 13 of the 18 years (72.2%) between 2008 and 2025 (Supplementary Table S2), with a mean annual increase of +5.16 (SD = 9.12). The largest year-over-year increase occurred in 2010 (+26.84), while the largest decrease occurred in 2011 (−9.25). Linear regression of *Δ*RSI over time showed no significant trend (slope = −0.23, R^2^ = 0.02, *p* = 0.56) (Figure 1c), and the ADF test confirmed that the first-difference series is stationary (*p* = 0.01). This indicates that the year-over-year changes fluctuate around a stable mean without a consistent upward or downward trajectory in the rate of growth. In contrast, cumulative *Δ*RSI demonstrated a strong linear increase over time (slope = 3.95, R^2^ = 0.93, *p* < 0.0001) (Figure 1d), reflecting the sustained long-term growth in public interest from a baseline RSI of 4.42 in 2007 to 81.00 in 2025—a net increase of +76.58 over 18 years.

For context, comparative RSI analysis was performed with two related, more general terms: “Kidney failure” and “Heart failure.” Interest in the specific term “Cardiorenal syndrome” was markedly lower, with an average RSI value below 1, compared to average RSI values of 22 for “Kidney failure” and 47 for “Heart failure” (Supplementary Figure S3).

### 3.2 Correlation between search trends and mortality

Data of mortality from the CDC WONDER database (2007–2020) demonstrated a sustained upward trend that paralleled the rise in search activity (Figure 2a). Linear regression confirmed a statistically significant increase in crude mortality rates over this period (slope = 0.95, R^2^ = 0.82, *p* < 0.001) (Figure 2b). However, the AD*F* test yielded a *p*-value of 0.42, with an ADF statistic of 6.48, exceeding the critical thresholds at the 1%, 5%, and 10% significance levels (−4.67, −3.37, and −2.80, respectively). Therefore, the null hypothesis of a unit root could not be rejected, indicating that the mortality time series was non-stationary and exhibited an underlying temporal trend.

**Figure 2:**
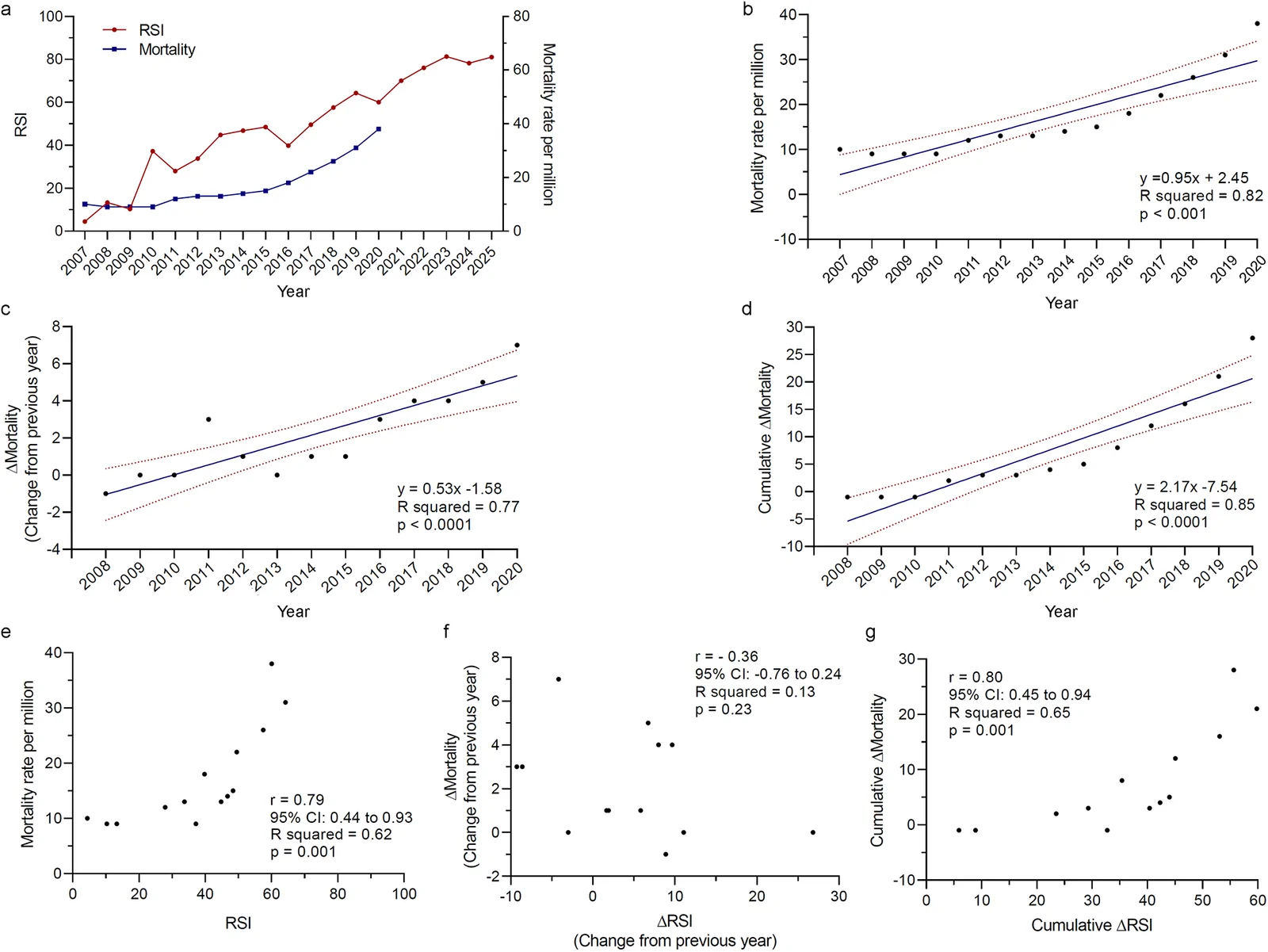
Temporal trends and associations between search activity and mortality in cardiorenal syndrome. **(a)** Trends in relative search interest (RSI) from Google Trends (2007–2025) compared with crude mortality rates from the CDC WONDER database (2007–2020). **(b)** Linear regression of crude mortality rates over time. **(c)** Linear regression of year-over-year changes in mortality (ΔMortality) over time, indicating accelerating mortality increase. **(d)** Linear regression of cumulative ΔMortality over time, demonstrating near-perfect linear growth in cumulative mortality burden. **(e)** Pearson correlation between raw annual RSI and raw annual mortality rates. **(f)** Pearson correlation between first-difference (year-over-year change) RSI and mortality, demonstrating no significant association after accounting for secular trends. **(g)** Pearson correlation between cumulative ΔRSI and cumulative ΔMortality rates, reflecting the parallel long-term growth trajectories of both metrics.

Analysis of year-over-year changes (*Δ*Mortality) revealed that mortality increased in 11 of the 13 years (84.6%) between 2008 and 2020 (Supplementary Table S3), with a mean annual increase of +2.15 (SD = 2.34). Linear regression of ΔMortality over time revealed a significant increasing trend (slope = 0.53, R^2^ = 0.77, *p* < 0.0001) (Figure 2c), indicating that the rate of mortality increase itself accelerated over the study period. Cumulative ΔMortality, representing the net change from the 2007 baseline, increased from −1.00 in 2008 to +28.00 in 2020, with a near-perfect linear relationship over time (slope = 2.17, R^2^ = 0.85, *p* < 0.0001) (Figure 2d), reflecting the sustained and accelerating growth in mortality burden.

At the national level, a strong positive correlation was observed between raw annual RSI values and raw annual mortality rates (r = 0.79, 95% CI: 0.44 to 0.93, *p* = 0.001), suggesting a temporal alignment between increasing public online interest and the rising disease burden (Figure 2e). However, given the non-stationarity of both time series (AD*F* test *p* = 0.98 for RSI and *p* = 0.42 for mortality), this raw correlation is potentially confounded by shared secular trends and should be interpreted with caution.

To address this limitation, we performed a first-difference (year-over-year change) analysis. The correlation between ΔRSI and *Δ*Mortality was r = −0.36 (95% CI: −0.76 to 0.24, *p* = 0.23) (Table 1, Figure 2f), indicating a negative but not statistically significant association between short-term fluctuations in search interest and mortality after accounting for secular trends. In the AR(1) regression model, the AR(1) coefficient was highly significant (*φ* = 0.89, 95% CI: 0.61 to 1.17, *p* < 0.0001), confirming the presence of strong, persistent autocorrelation in the residuals. After accounting for this autocorrelation, the association between RSI and mortality was not statistically significant (*β* = 1.58, 95% CI: −0.47 to 3.63, *p* = 0.11). The Durbin-Watson statistic improved from 0.85 in the ordinary least squares (OLS) model to 1.94 in the AR(1) model, indicating that the autoregressive structure successfully captured the autocorrelation (Table 1). These findings are consistent with the first-difference analysis and confirm that the significant raw correlation (r = 0.79, *p* = 0.001) was confounded by shared secular trends and autocorrelation.

**Table 1:** Sensitivity analysis of correlation between relative search interest and mortality across various ICD-10 113 cause of death code using “AND” logic.

Cause of death code combination	*Δ*RSI and *Δ*Mortality (First-difference analysis)^a^	Cumulative *Δ*RSI and cumulative *Δ*Mortality^a^	RSI and mortality (Raw series)^a^	RSI and mortality			
				DW-test from OLS residuals^b^	AR(1) coefficient *φ*^c^	AR(1) modeling^d^	DW-test in AR(1) modeling^e^
I13	r = −0.36, 95%CI: −0.76 to 0.24 *p* = 0.23	r = 0.80 95% CI: 0.45 to 0.94 *p* = 0.001	r = 0.79 95% CI: 0.44 to 0.93 *p* = 0.001	d = 0.85 95%CI: 0.00 to 1.90	φ=0.89 95%CI: 0.61 to 1.17 *p* < 0.001	*β*=1.58 95% CI: −0.47 to 3.63 *p* = 0.11	1.94
I13&I50	r = −0.32 95%CI: −0.74 to 0.28 *p* = 0.28	r = 0.80 95% CI: 0.45 to 0.94 *p* = 0.001	r = 0.85 95% CI: 0.58 to 0.95 *p* < 0.001	d = 0.79 95%CI: 0.00 to 1.84	φ=0.87 95%CI: 0.57 to 1.17 *p* < 0.001	β=2.42 95% CI: −0.64 to 5.48 *p* = 0.11	1.92
I13&N17-N19	r = −0.42 95%CI: −0.79 to 0.17 *p* = 0.15	r = 0.07 95% CI: −0.50 to 0.60 *p* = 0.82	r = 0.62 95% CI: 0.13 to 0.87 *p* = 0.019	d = 0.92 95%CI: 0.00 to 1.97	φ=0.92 95%CI: 0.68 to 1.16 *p* < 0.001	β=2.91 95% CI: −0.53 to 6.35 *p* = 0.09	1.96
I13& I50&N17-N19^f^	r = −0.44 95%CI: −0.79 to 0.15 *p* = 0.14	r = 0.79 95% CI: 0.42 to 0.93 *p* = 0.001	r = 0.78 95% CI: 0.43 to 0.93 *p* = 0.001	d = 0.88 95%CI: 0.00 to 1.93	φ=0.88 95%CI: 0.58 to 1.18 *p* < 0.001	β=1.82 95% CI: −0.24 to 3.88 *p* = 0.08	1.93

RSI, relative search interest; DW, Durbin-Watson; OLS, ordinary least squares; AR, autoregressive.

a Pearson correlation.

b Across all four ICD-10 definitions, the upper bound of the 95% confidence interval for the DW statistic does not reach 2.0 (ranging from 1.84 to 1.97). This confirms that all series exhibit significant positive autocorrelation, justifying the use of AR(1) modeling for all definitions.

c All AR(1) coefficients (φ) are significant at *p* < 0.001, confirming the presence of strong, persistent autocorrelation in the residuals.

d After accounting for this autocorrelation, the association between RSI and mortality was not statistically significant across all definitions.

e The DW statistics improved from below 0.92 in the unadjusted OLS models to between 1.92 and 1.96 in the AR(1) models, indicating successful correction for autocorrelation.

f This combination is equivalent to “(I13 OR I50) AND N17–N19” because CDC WONDER's Boolean query interface allows “AND” logic between only two code groups.

CRS is conventionally classified into five types based on the primary organ involved, acuity, and direction of organ interaction. No dedicated ICD-10 code exists that comprehensively captures exact or all types of CRS-related mortality. ICD-10 code I13.x (hypertensive heart and renal disease) primarily represents a chronic heart–kidney phenotype, making it most relevant to CRS type 2 and 4 or overlapping chronic cardiorenal disease (7). However, it would poorly capture type 1 and type 3 CRS, where acute hear failure (I50.x) and AKI (N17.x) are central, as well as type 5, where the underlying systemic disorder may be coded as the primary cause of death. In the ICD-10 113 mortality cause list, AKI and/or acute renal failure (N17), CKD (N18) and other unspecified kidney failure (N19) are combined into the N17-N19 group, which explicitly defines kidney failure. To address this limitation and to ensure that our findings are robust to the choice of ICD-10 definition, we performed comprehensive sensitivity analyses using alternative ICD-10 definitions that combine I13.x with heart failure (I50.x), CKD (N18.x–N19.x), and AKI (N17.x) codes (Supplementary Table S4).

Using the first-difference approach, correlations between *Δ*RSI and *Δ*Mortality ranged from r = −0.32 to r = −0.44, with all *p*-values remaining non-significant (*p* = 0.14 to 0.28) (Table 1). To further account for temporal autocorrelation, we performed AR(1) regression analyses for each ICD-10 mortality definition (Table 1). Across all definitions, the AR(1) coefficients were not statistically significant: I13 mortality (*β* = 1.58, 95% CI: −0.47 to 3.63, *p* = 0.11), I13&I50 mortality (*β* = 2.42, 95% CI: −0.64 to 5.48, *p* = 0.11), I13&N17-N19 mortality (*β* = 2.91, 95% CI: −0.53 to 6.35, *p* = 0.09), and comprehensive definition incorporating I13&I50&N17-N19 (*β* = 1.82, 95% CI: −0.24 to 3.88, *p* = 0.08). All AR(1) coefficients (*φ*) ranged from 0.87 to 0.92 (all *p* < 0.001), confirming strong autocorrelation in all series. The Durbin-Watson statistics improved from below 0.92 in the unadjusted OLS models to between 1.92 and 1.96 in the AR(1) models, indicating successful correction for autocorrelation. These consistent findings confirm that the absence of a significant short-term association between search interest and mortality is robust to the choice of ICD-10 definition.

To capture a broader spectrum of CRS-related mortality, we applied several expanded ICD-10 code combinations, including I13/I50, I13/N17–N19, and I13/I50/N17–N19 (Supplementary Table S5 and S6). AR(1) modeling demonstrated significant associations between RSI and mortality when mortality was defined using I13/I50 or I13/I50/N17–N19. These findings suggest that the observed relationship between public search interest and mortality is sensitive to the ICD-10 definition used to identify CRS-related deaths. However, these broader definitions may have reduced specificity for CRS and potentially overestimated CRS-related mortality by capturing deaths attributable to cardiovascular or kidney disease that were not specifically related to CRS. Therefore, the significant associations observed with these broader definitions should be interpreted cautiously. In contrast to the first-difference analysis and AR(1) modeling, analysis of cumulative changes revealed a strong positive correlation between cumulative *Δ*RSI and cumulative *Δ*Mortality (r = 0.80, 95% CI: 0.45 to 0.94, *p* = 0.001) (Table 1, Figure 2g). This association reflects the parallel long-term growth trajectories of both metrics, with cumulative ΔRSI increasing from +8.91 in 2008 to +55.66 in 2020 and cumulative *Δ*Mortality increasing from −1 to +28.00 over the same period. Similarly, the comprehensive definition (I13&I50 & N17–N19) also demonstrated a strong correlation between cumulative changes (r = 0.79, 95% CI: 0.42 to 0.93, *p* = 0.001) (Table 1). This consistency across definitions indicates that the parallel long-term growth trajectories are not an artifact of the I13.x alone definition.

### 3.3 Regional disparities in search interest

All significant geographic variation in search interest was observed across the United States (Figure 3a, Supplementary Table S3). West Virginia exhibited the highest relative search activity (RSI = 100). States were grouped into tiers of descending interest: twelve states (e.g., Vermont, Minnesota) showed high interest (RSI = 60), twenty-seven states (e.g., South Dakota, Kentucky) showed moderate interest (RSI = 40), and eleven states (e.g., Wyoming, Georgia, Texas) showed the lowest relative search activity (RSI = 20).

**Figure 3:**
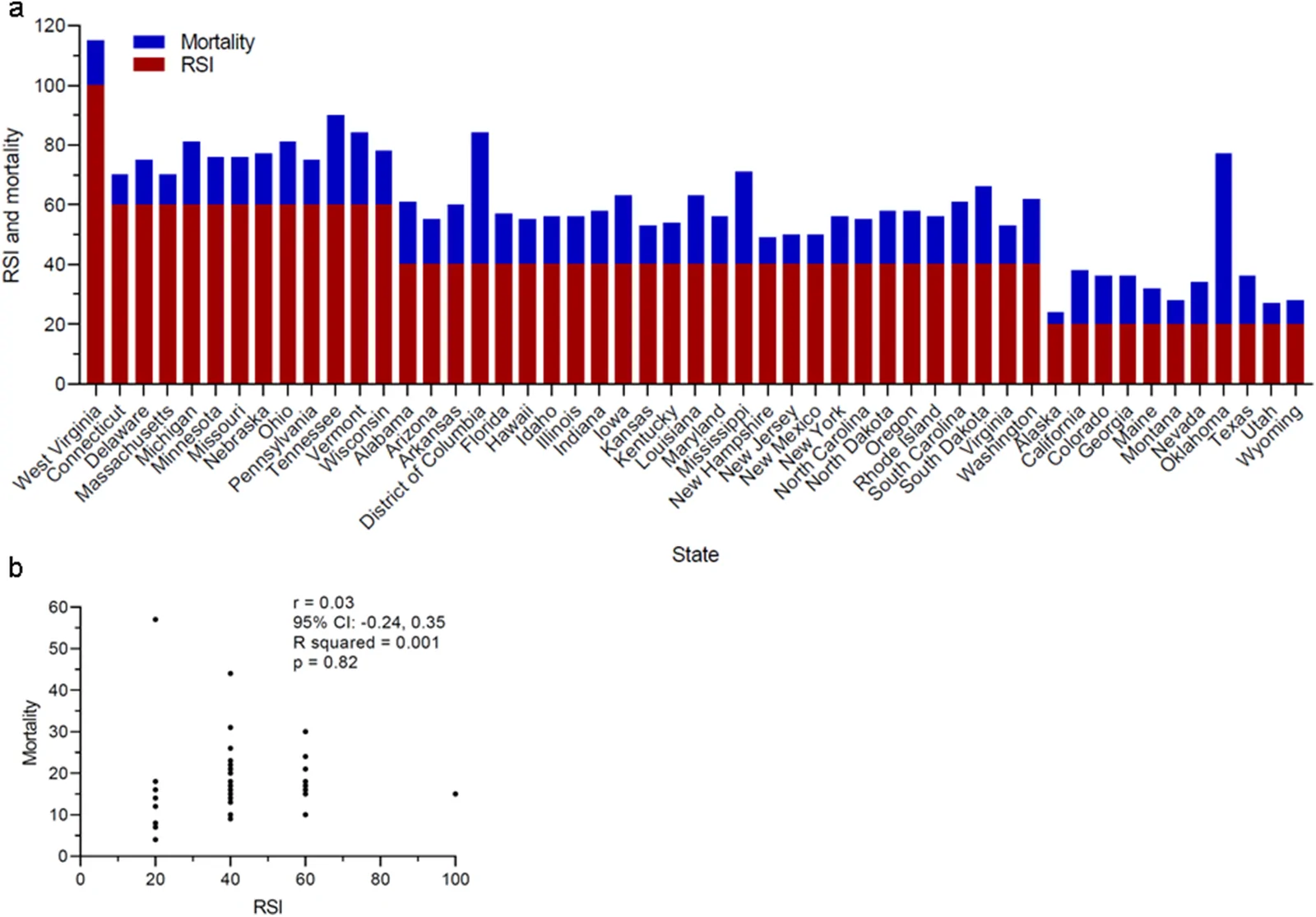
Regional variation in search activity for cardiorenal syndrome. **(a)** The relative search interest (RSI) and mortality rate (per million) across different states. **(b)** Pearson correlation analysis indicating no significant relationship between RSI and state-level mortality rates.

State-level mortality rates from 2007 to 2020 varied widely, ranging from 4 to 57 deaths per million. West Virginia, with the highest RSI, had a relatively low mortality rate of 15 per million. Conversely, Oklahoma, with the lowest RSI (20), experienced the highest mortality rate (57 per million).

Pearson correlation analysis formally confirmed the lack of a significant association between state-level RSI and mortality rates (r = 0.03, 95% CI: 0.24 to 0.35, *p* = 0.82) (Figure 3b). These findings reveal a disconnect between regional disease burden and public information-seeking behavior, highlighting a critical need for targeted awareness campaigns, particularly in high-mortality, low-search-activity regions.

We retrieved age-adjusted mortality rates (standardized to the 2000 U.S. standard population) for ICD-10 code I13.x from the CDC WONDER database for the period 2007–2020 (Supplementary Table S7). This adjustment removes the confounding effect of differing state age structures, allowing for a more valid comparison of mortality risk across states. The results a slightly negative correlation (r = −0.09, 95% CI: −0.36 to 0.19, *p* = 0.52), which remains non-significant (Supplementary Figure S4). This confirms that the lack of association between state-level RSI and mortality is robust to age adjustment.

A crude, unadjusted correlation between RSI and mortality may be misleading, because both variables are likely influenced by underlying socioeconomic and healthcare access factors—such as income, education, and insurance coverage (Supplementary Table S7)—that could confound, suppress, or artificially inflate the observed relationship; therefore, adjusted analyses have been performed using ordinary least-squares linear regression to isolate the independent contribution of RSI, if any, and to determine whether the association persists beyond these key demographic and structural determinants of health.

In linear regression, analyses (Table 2), unadjusted model indicates that each 1-unit increase in RSI was associated with a 0.047-unit lower age-adjusted mortality, but the confidence interval is wide and crosses zero (*β* = −0.047, 95% CI −0.194 to 0.099; *p* = 0.52). The association remained nonsignificant after separate adjustment for median family income (*β* = −0.049, *p* = 0.51), educational attainment (*β* = −0.046, *p* = 0.53), or insurance coverage (*β* = 0.017, *p* = 0.84). Results were similarly nonsignificant in models incorporating pairs of socioeconomic covariates (*β* ranging from −0.047 to 0.060, *p* ranging from 0.50 to 0.78). In the fully adjusted model including median family income, educational attainment, and insurance coverage, RSI remained unassociated with age-adjusted mortality (*β* = 0.029, 95% CI −0.149 to 0.207; *p* = 0.75).

**Table 2:** Linear regression analysis for correlation between RSI and age-adjusted mortality at state level.

Model	Adjustment variables	RSI β	95% CI	*p* value	R^2^
Unadjusted	None	−0.047	−0.194 to 0.099	0.52	0.009
1 variable	Income	−0.049	−0.197 to 0.098	0.51	0.019
	Education	−0.046	−0.195 to 0.102	0.53	0.011
	Insurance rate	+0.017	−0.151 to 0.184	0.84	0.054
2 variables	Income + Education	−0.047	−0.190 to 0.095	0.51	0.110
	Income + Insurance	+0.026	−0.159 to 0.211	0.78	0.055
	Education + Insurance	+0.060	−0.117 to 0.238	0.50	0.093
3 variables	Income + Education + Insurance	+0.029	−0.149 to 0.207	0.75	0.147

## 4 Discussion

This study demonstrates a significant upward trajectory in U.S. public interest in CRS over the past two decades, as measured by online search activity. This trend closely parallels a concurrent national increase in mortality with hypertensive heart and CKD as the underlying cause, yet reveals pronounced geographic disparities in awareness, underscoring a complex and uneven information landscape.

The sustained rise in search interest for “Cardiorenal syndrome” is a likely reflection of the growing epidemiological burden of its underlying components—heart failure and CKD (6, 7, 10, 11). As the burden of these conditions continues to grow, it is reasonable to expect increased information-seeking behavior among populations. As the prevalence of these conditions increases, public engagement through information-seeking represents a natural, albeit passive, response. The correlative relationship observed at the national level between search interest and mortality further suggests that aggregated digital interest may serve as a novel signal for tracking long-term disease burden, aligning with established principles of infodemiology (16–19). However, several important caveats regarding the interpretation of our findings merit discussion.

First, while the raw correlation between RSI and mortality was strong across all ICD-10 definitions (r = 0.62 to 0.85, all *p* < 0.05), this association was largely driven by the shared secular trends in both series. After applying a first-difference transformation to remove these trends, the correlation between *Δ*RSI and *Δ*Mortality was not statistically significant across any definition, with correlations ranging from r = −0.32 to r = −0.44 (all *p* = 0.14 to 0.28). This finding was further confirmed by an autoregressive AR(1) model, which explicitly corrected for autocorrelation and yielded a non-significant association across all definitions (*β* = 1.58 to 2.91, all *p* = 0.08 to 0.11). Importantly, these results were consistent regardless of whether mortality was defined using the I13.x alone or its combinations incorporating heart failure (I50.x), CKD (N18.x–N19.x), and AKI (N17.x) codes. The consistency of these findings across multiple ICD-10 definitions confirms that the absence of a significant short-term association between search interest and mortality is robust to the choice of mortality definition. Collectively, these results substantially temper any claims regarding the utility of Google Trends as a near-real-time disease surveillance tool for CRS. Short-term fluctuations in public search interest do not appear to reliably track contemporaneous changes in mortality, and thus our data do not support the use of Google Trends for predicting or monitoring short-term epidemiological trends.

Second, the strong correlation between cumulative ΔRSI and cumulative ΔMortality across various mortality definitions (r = 0.79 to 0.80, all *p* = 0.001) reflects the parallel long-term growth trajectories of both metrics rather than a dynamic, short-term association. We interpret this alignment as evidence that public interest and disease burden have increased in tandem over the study period, likely driven by overlapping but independent factors—including the rising prevalence of heart failure and CKD, increased media attention to cardiorenal health, and greater efforts in clinical and patient education. Notably, the correlation between ΔRSI and ΔMortality for the I13&N17-N19 definition was r = 0.07 (95% CI: −0.50 to 0.61, *p* = 0.82), which is essentially zero and not statistically significant. This definition incorporates AKI (N17.x) and CKD (N18.x–N19.x) codes while excluding heart failure codes (I50.x), and thus primarily captures kidney-predominant CRS mortality. The near-zero correlation likely reflects the episodic nature of AKI-related deaths and the lower public awareness of CKD compared to heart failure.

Third, the sensitivity of the association to the mortality definition highlights an important methodological challenge in studying CRS at the population level. Broader ICD-10 definitions may better capture the heterogeneous cardiorenal spectrum and reduce under-ascertainment, but they do so at the expense of specificity, potentially including deaths not directly attributable to CRS. Thus, the significant associations observed with broader definitions may reflect improved capture of CRS burden, misclassification from inclusion of related cardiovascular and kidney disease, or both. The lack of a standardized ICD-based definition of CRS mortality therefore remains an important limitation when interpreting population-level associations.

Additionally, the differential trends observed between ΔRSI (stable growth rate) and ΔMortality (accelerating growth rate) suggest that public interest may not be keeping pace with the accelerating disease burden, highlighting a potential awareness gap that warrants targeted public health interventions. While RSI demonstrated a stable growth rate over time (slope = −0.23, *p* = 0.56), mortality exhibited a significant accelerating increase (slope = 0.53, *p* < 0.0001 for *Δ*Mortality; slope = 2.17, R^2^ = 0.85, *p* < 0.0001 for cumulative ΔMortality). This disparity is particularly concerning given that CRS mortality—even when measured using the I13.x definition—rose from 9.9 to 37.6 per million (a 101% increase) between 2007 and 2020, and when measured using the comprehensive definition, the increase was even more pronounced, rising from 5.2 to 24.9 per million—a 379% surge. The accelerating mortality burden underscores the urgent need for improved prevention, early detection, and management strategies for CRS, particularly as the population ages and risk factors become more prevalent.

Notably, a comparative analysis of other major English-speaking nations (Australia, Canada, the United Kingdom, and New Zealand) revealed a similar, though less pronounced, upward trend in search activity. While differences in population size must be considered in cross-national comparisons, this pattern may indicate a nascent but growing global awareness of CRS. Domestically, however, search interest for CRS remains markedly lower than for its more recognized constituent conditions, “Kidney failure” and “Heart failure.” This disparity likely stems from the syndrome's lower overall incidence and its status as a specialized medical term, potentially less accessible to the general public. This is concerning, given that an estimated 36% of U.S. adults possess only basic or below-basic health literacy, which may limit proactive recognition and understanding of complex conditions like CRS (22).

The significant state-level variation in search interest was not associated with corresponding differences in mortality. This null association persisted after adjustment for median family income, educational attainment, and insurance coverage, suggesting that regional variation in online public interest in CRS does not closely parallel the geographic distribution of its estimated mortality burden. This discordance was evident in individual states; for example, West Virginia had the highest search interest despite a relatively moderate mortality rate, whereas Oklahoma demonstrated the opposite pattern, with the highest mortality rate but relatively low search interest. These findings suggest a potential disconnect between population-level disease burden and online information-seeking behavior. However, public engagement with CRS is likely multifactorial and cannot be explained by disease burden or the socioeconomic factors examined in this study alone. Unmeasured factors, including regional differences in disease recognition and clinical diagnosis, health literacy, patient–provider communication, access to health information, healthcare utilization, and local or media-driven awareness, may substantially influence online search behavior. Thus, geographic search patterns may reflect a complex interplay of clinical, healthcare, and informational factors rather than serving as a direct surrogate for underlying disease burden. Notably, rather than implying a direct relationship between awareness (as measured by search interest) and mortality, this study aims to identify geographic regions where awareness is potentially discordant with mortality burden. Our findings suggest that regions with low search activity but high mortality may represent priority areas for targeted public health interventions, regardless of whether the association is confounded or causal.

These findings carry direct implications for public health strategy. The overall low volume of CRS-specific searches, despite its rising clinical significance, signals a substantial awareness gap. Bridging this gap requires proactive, targeted educational campaigns that move beyond passive information availability. Messaging should be tailored to address local barriers, simplified to overcome literacy challenges, and disseminated through channels accessible to high-risk demographics. In regions with low search activity, interventions might focus on foundational education about CRS symptoms and risk factors, potentially integrated into existing heart failure and CKD management programs.

This study has several limitations. First, the analysis relied on a single, precise English-language search term, which may not capture the full spectrum of public queries, as individuals may use lay terminology or search for specific symptoms instead. This also limits the generalizability of findings to non-English-speaking populations within and beyond the United States. Second, the normalized nature of Google Trends data precludes analysis of absolute search volumes and obscures the demographic profile of searchers, making it difficult to distinguish between queries from patients, caregivers, healthcare professionals, or students. Third, this study is limited by the lack of a specific ICD-10 code for CRS-related mortality; deaths were instead captured using codes for the underlying condition (primarily I13.x, hypertensive heart and CKD). Although we tested multiple coding definitions, our mortality estimates may either under- or overrepresent the true CRS burden, and potential outcome misclassification could affect the observed relationship between search trends and mortality. Furthermore, while we adjusted for age, income, education, and insurance coverage in state-level analysis, residual confounding by unmeasured factors such as physician awareness, health literacy, or regional coding practices may still influence both mortality and online search behavior. Finally, our trend-adjusted correlation is a more appropriate, albeit still exploratory, approach for our dataset. Nevertheless, the relatively small number of annual observations (with *n* = 14) precludes the use of more sophisticated time-series models (e.g., ARIMA or vector autoregression). As a proof-of-concept analysis, this study highlights the need for future studies with longer time series or higher-frequency (e.g., weekly or monthly) data to enable more powerful and complex time-series modeling.

## 5 Conclusions

This infodemiological study documents a rising but uneven public awareness of CRS in the United States. While digital search trends have increased in parallel with the syndrome's growing burden at the national level, the absence of a significant short-term association between changes in search interest and changes in mortality tempers claims regarding the utility of Google Trends as a real-time disease surveillance tool for CRS. The parallel long-term trends suggest that public awareness and disease burden have increased in tandem, likely driven by overlapping factors including rising disease prevalence and increased media attention. But the observed relationship between public interest and CRS-related mortality was sensitive to the ICD-10 definition used, underscoring the need for standardized and validated criteria to define CRS mortality in population-based studies.

Significant regional disparities highlight gaps where public knowledge lags behind clinical need, with states such as Oklahoma exhibiting low search interest despite high mortality burden. Utilizing these insights can guide the development of more effective, geographically and demographically tailored public health communications to improve early recognition and management of this consequential syndrome.

This analysis establishes a foundation for future work; however, to build a more comprehensive understanding of public awareness, subsequent research must adopt a multi-modal approach. This should include the use of diverse keyword sets and data from other social media or health forums, complemented by qualitative investigations into user intent. Most critically, longitudinal studies are needed to measure the real-world efficacy of awareness campaigns by assessing their influence on both search behavior and, ultimately, clinical endpoints.

## Data Availability

The original contributions presented in the study are included in the article/Supplementary Material, further inquiries can be directed to the corresponding author.
