# Supplementary material for: Growing public interest and regional disparities of cardiorenal syndrome in the United States
Source: Front Digit Health. 2026 Sep 15;8:1803510. doi: 10.3389/fdgth.2026.1803510 (PMC13619914; doi:10.3389/fdgth.2026.1803510)
Supplement: Supplementary file 1 [file Datasheet1.pdf]

Supplementary Material

1 Supplementary Figures

1.1 Supplementary Figure 1

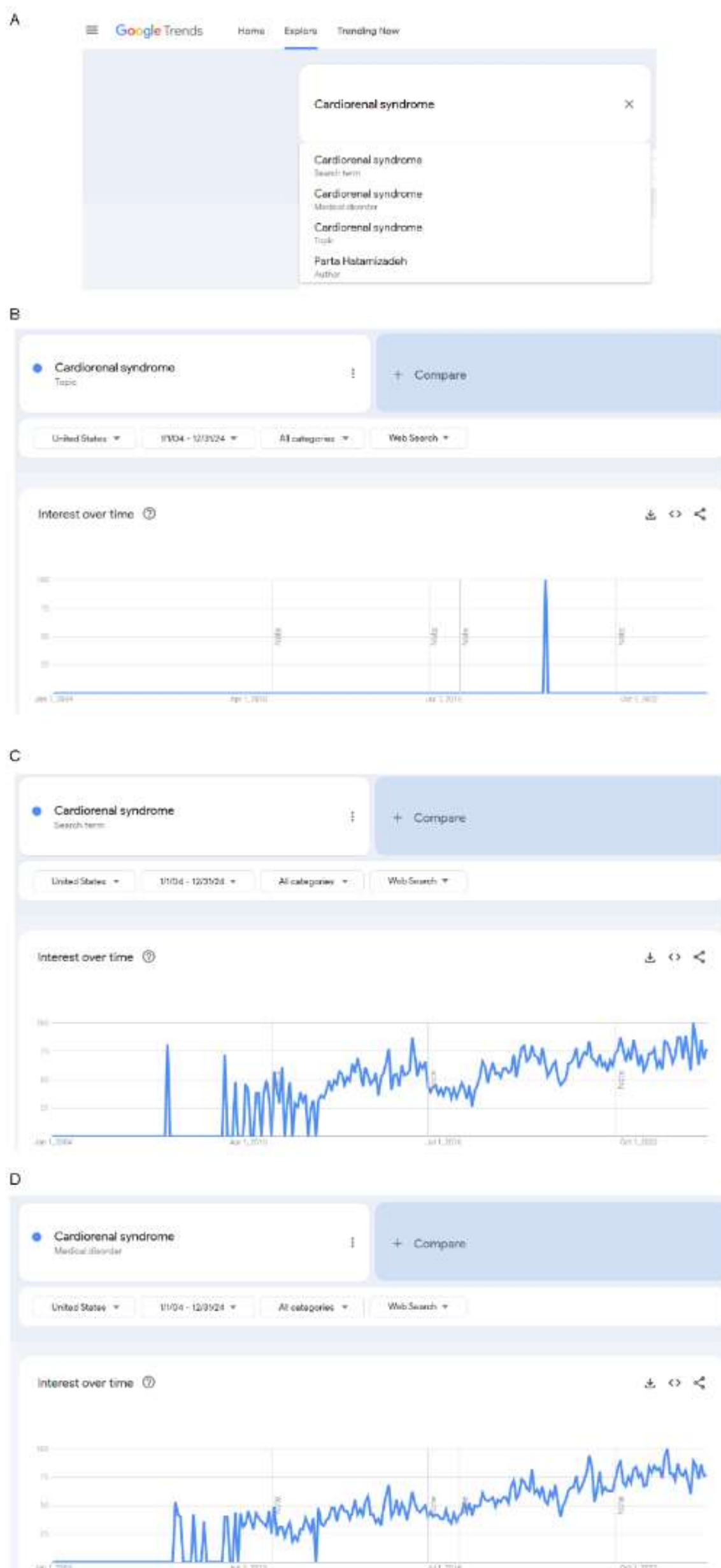

Supplementary Figure 1. Google Trends search activity for cardiorenal syndrome across different categories. (A) Available search categories for "Cardiorenal Syndrome" in Google Trends. (B) Search activity under the Topic category. (C) Search activity under the Search Term category. (D) Search activity under the Medical Disorder category.

1.2 Supplementary Figure 2

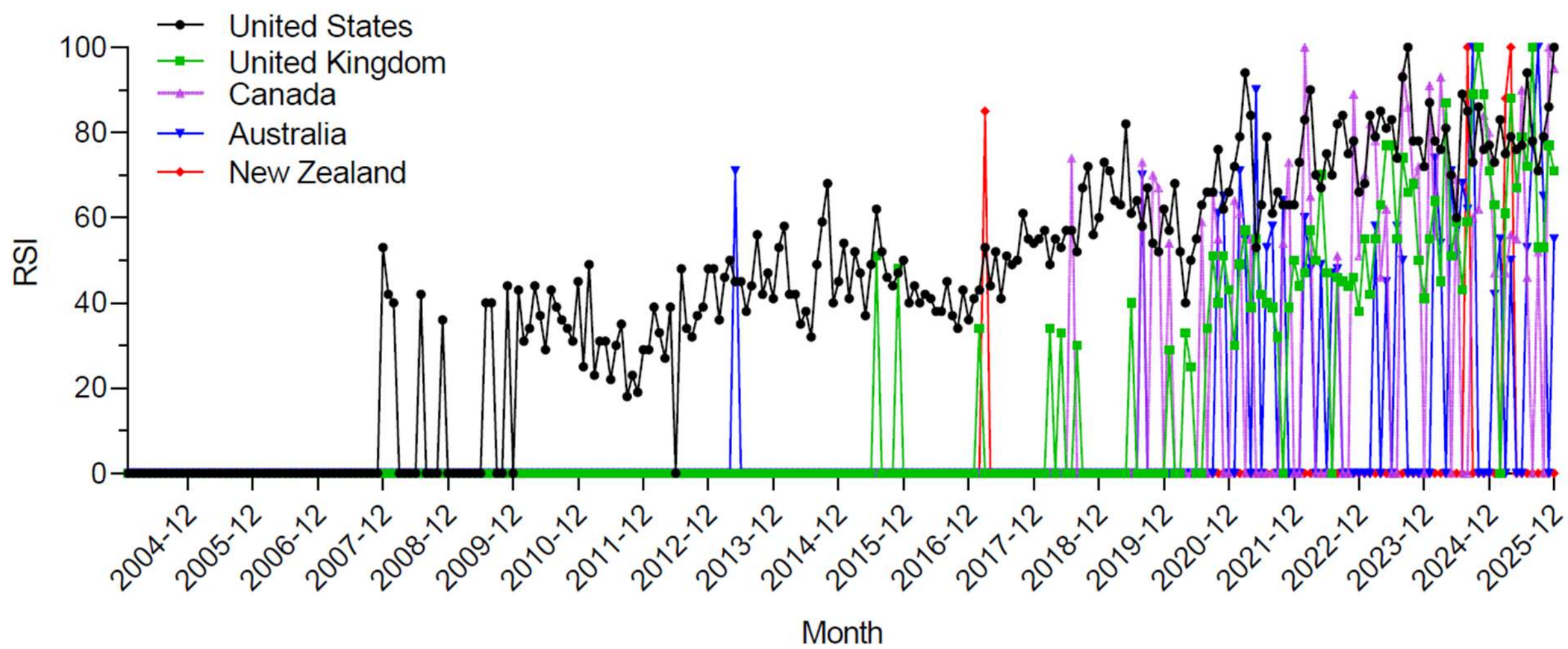

Supplementary Figure 2. Monthly relative search activity for cardiorenal syndrome on Google Trends. The relative search interest (RSI) index for cardiorenal syndrome under the Medical Disorder category was plotted over the past two decades based on monthly data retrieved from Google Trends.

1.3 Supplementary Figure 3

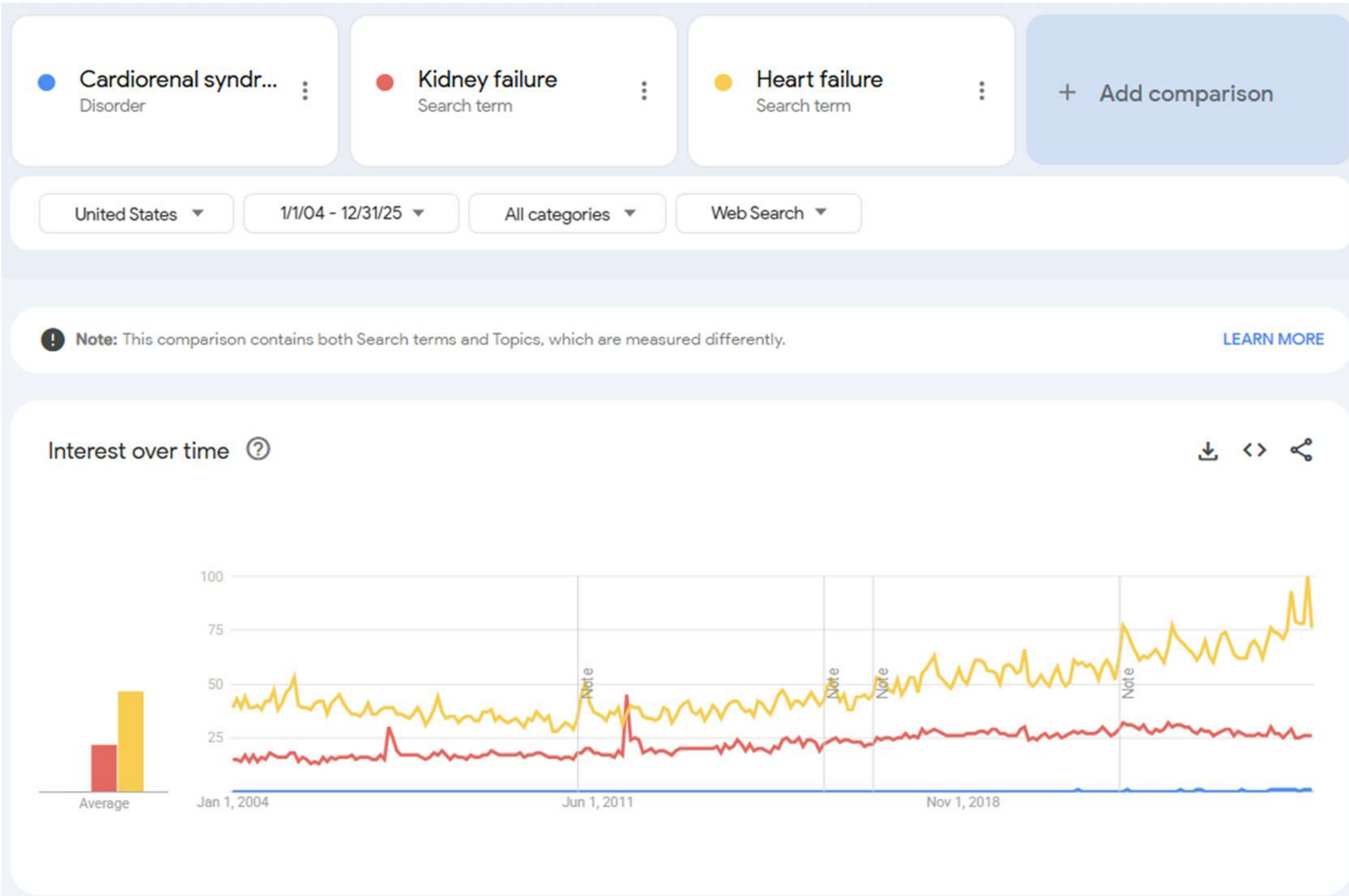

Supplementary Figure 3. Google Trends analysis on cardiorenal syndrome, kidney failure, and heart failure.

1.4 Supplementary Figure 4

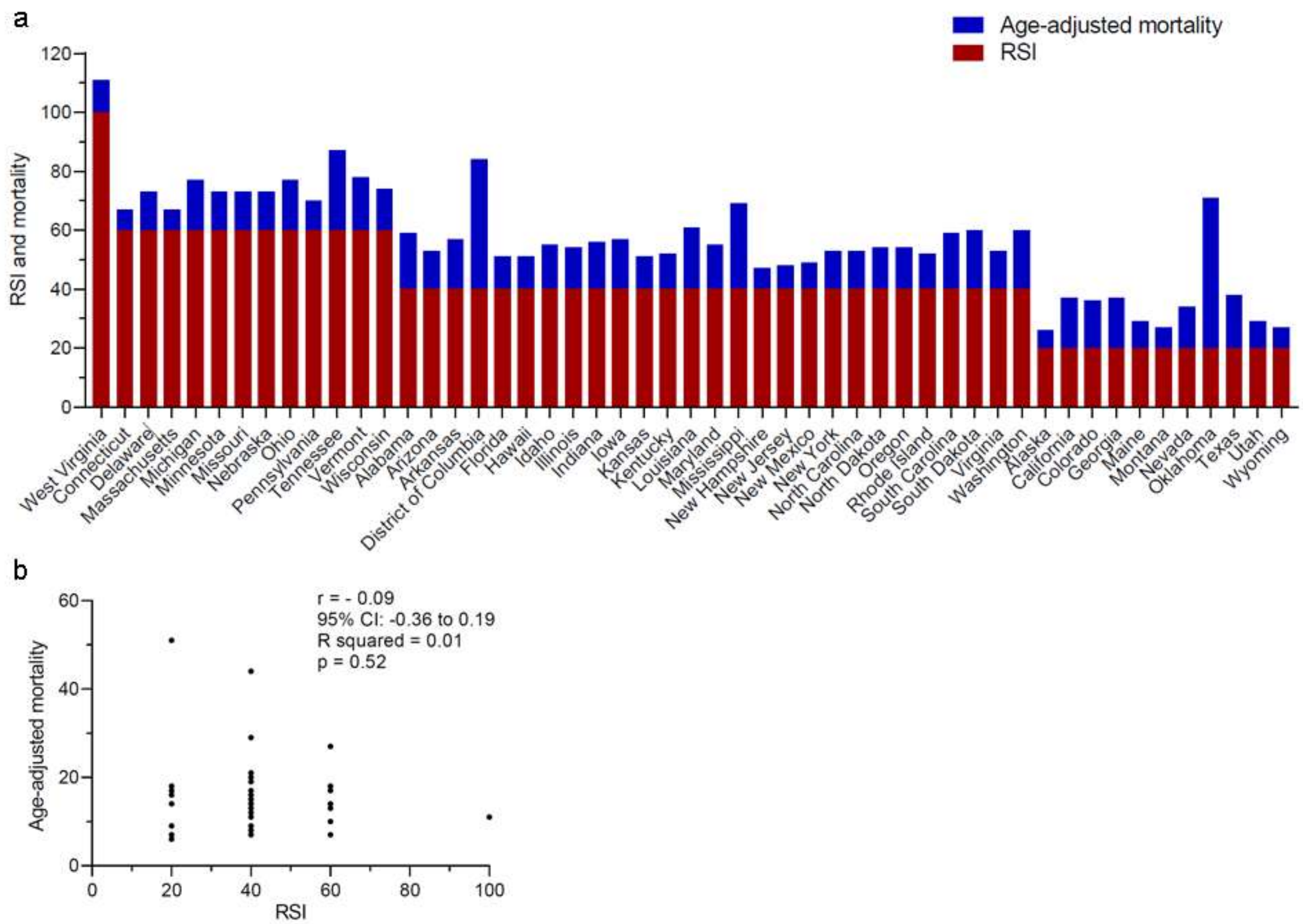

Supplementary Figure 4. Regional variation in search activity and age-adjusted mortality for cardiorenal syndrome. (a). The relative search interest (RSI) and age-adjusted mortality rate (per million) across different states. (b). Pearson correlation analysis indicating no significant relationship between RSI and state-level age-adjusted mortality rates.

2 Supplementary Tables

2.1 Supplementary Table 1

Table S1. Monthly-based search activity for cardiorenal syndrome from January 1, 2004, to December 31, 2025, obtained from Google Trends.

Month	United States	United Kingdom	Canada	Australia	New Zealand
2004-01	0	0	0	0	0
2004-02	0	0	0	0	0
2004-03	0	0	0	0	0
2004-04	0	0	0	0	0
2004-05	0	0	0	0	0
2004-06	0	0	0	0	0
2004-07	0	0	0	0	0
2004-08	0	0	0	0	0
2004-09	0	0	0	0	0
2004-10	0	0	0	0	0
2004-11	0	0	0	0	0
2004-12	0	0	0	0	0
2005-01	0	0	0	0	0
2005-02	0	0	0	0	0
2005-03	0	0	0	0	0
2005-04	0	0	0	0	0
2005-05	0	0	0	0	0
2005-06	0	0	0	0	0
2005-07	0	0	0	0	0
2005-08	0	0	0	0	0
2005-09	0	0	0	0	0
2005-10	0	0	0	0	0
2005-11	0	0	0	0	0
2005-12	0	0	0	0	0
2006-01	0	0	0	0	0
2006-02	0	0	0	0	0
2006-03	0	0	0	0	0
2006-04	0	0	0	0	0
2006-05	0	0	0	0	0
2006-06	0	0	0	0	0

2006-07	0	0	0	0	0
2006-08	0	0	0	0	0
2006-09	0	0	0	0	0
2006-10	0	0	0	0	0
2006-11	0	0	0	0	0
2006-12	0	0	0	0	0
2007-01	0	0	0	0	0
2007-02	0	0	0	0	0
2007-03	0	0	0	0	0
2007-04	0	0	0	0	0
2007-05	0	0	0	0	0
2007-06	0	0	0	0	0
2007-07	0	0	0	0	0
2007-08	0	0	0	0	0
2007-09	0	0	0	0	0
2007-10	0	0	0	0	0
2007-11	0	0	0	0	0
2007-12	53	0	0	0	0
2008-01	42	0	0	0	0
2008-02	40	0	0	0	0
2008-03	0	0	0	0	0
2008-04	0	0	0	0	0
2008-05	0	0	0	0	0
2008-06	0	0	0	0	0
2008-07	42	0	0	0	0
2008-08	0	0	0	0	0
2008-09	0	0	0	0	0
2008-10	0	0	0	0	0
2008-11	36	0	0	0	0
2008-12	0	0	0	0	0
2009-01	0	0	0	0	0
2009-02	0	0	0	0	0
2009-03	0	0	0	0	0
2009-04	0	0	0	0	0
2009-05	0	0	0	0	0
2009-06	0	0	0	0	0
2009-07	40	0	0	0	0

2009-08	40	0	0	0	0
2009-09	0	0	0	0	0
2009-10	0	0	0	0	0
2009-11	44	0	0	0	0
2009-12	0	0	0	0	0
2010-01	43	0	0	0	0
2010-02	31	0	0	0	0
2010-03	34	0	0	0	0
2010-04	44	0	0	0	0
2010-05	37	0	0	0	0
2010-06	29	0	0	0	0
2010-07	43	0	0	0	0
2010-08	39	0	0	0	0
2010-09	36	0	0	0	0
2010-10	34	0	0	0	0
2010-11	31	0	0	0	0
2010-12	45	0	0	0	0
2011-01	25	0	0	0	0
2011-02	49	0	0	0	0
2011-03	23	0	0	0	0
2011-04	31	0	0	0	0
2011-05	31	0	0	0	0
2011-06	22	0	0	0	0
2011-07	30	0	0	0	0
2011-08	35	0	0	0	0
2011-09	18	0	0	0	0
2011-10	23	0	0	0	0
2011-11	19	0	0	0	0
2011-12	29	0	0	0	0
2012-01	29	0	0	0	0
2012-02	39	0	0	0	0
2012-03	33	0	0	0	0
2012-04	27	0	0	0	0
2012-05	39	0	0	0	0
2012-06	0	0	0	0	0
2012-07	48	0	0	0	0
2012-08	34	0	0	0	0
2012-09	32	0	0	0	0

2012-10	37	0	0	0	0
2012-11	39	0	0	0	0
2012-12	48	0	0	0	0
2013-01	48	0	0	0	0
2013-02	36	0	0	0	0
2013-03	46	0	0	0	0
2013-04	50	0	0	0	0
2013-05	45	0	0	71	0
2013-06	45	0	0	0	0
2013-07	38	0	0	0	0
2013-08	44	0	0	0	0
2013-09	56	0	0	0	0
2013-10	42	0	0	0	0
2013-11	47	0	0	0	0
2013-12	41	0	0	0	0
2014-01	53	0	0	0	0
2014-02	58	0	0	0	0
2014-03	42	0	0	0	0
2014-04	42	0	0	0	0
2014-05	35	0	0	0	0
2014-06	38	0	0	0	0
2014-07	32	0	0	0	0
2014-08	49	0	0	0	0
2014-09	59	0	0	0	0
2014-10	68	0	0	0	0
2014-11	40	0	0	0	0
2014-12	45	0	0	0	0
2015-01	54	0	0	0	0
2015-02	41	0	0	0	0
2015-03	52	0	0	0	0
2015-04	47	0	0	0	0
2015-05	37	0	0	0	0
2015-06	49	0	0	0	0
2015-07	62	51	0	0	0
2015-08	52	0	0	0	0
2015-09	46	0	0	0	0
2015-10	44	0	0	0	0

2015-11	47	48	0	0	0
2015-12	50	0	0	0	0
2016-01	40	0	0	0	0
2016-02	44	0	0	0	0
2016-03	40	0	0	0	0
2016-04	42	0	0	0	0
2016-05	41	0	0	0	0
2016-06	38	0	0	0	0
2016-07	38	0	0	0	0
2016-08	45	0	0	0	0
2016-09	37	0	0	0	0
2016-10	34	0	0	0	0
2016-11	43	0	0	0	0
2016-12	36	0	0	0	0
2017-01	41	0	0	0	0
2017-02	43	34	0	0	0
2017-03	53	0	0	0	85
2017-04	44	0	0	0	0
2017-05	52	0	0	0	0
2017-06	41	0	0	0	0
2017-07	51	0	0	0	0
2017-08	49	0	0	0	0
2017-09	50	0	0	0	0
2017-10	61	0	0	0	0
2017-11	55	0	0	0	0
2017-12	54	0	0	0	0
2018-01	55	0	0	0	0
2018-02	57	0	0	0	0
2018-03	49	34	0	0	0
2018-04	55	0	0	0	0
2018-05	53	33	0	0	0
2018-06	57	0	0	0	0
2018-07	57	0	74	0	0
2018-08	52	30	0	0	0
2018-09	67	0	0	0	0
2018-10	72	0	0	0	0
2018-11	56	0	0	0	0
2018-12	60	0	0	0	0
2019-01	73	0	0	0	0

2019-02	71	0	0	0	0
2019-03	64	0	0	0	0
2019-04	63	0	0	0	0
2019-05	82	0	0	0	0
2019-06	61	40	0	0	0
2019-07	64	0	0	0	0
2019-08	58	0	73	70	0
2019-09	67	0	0	0	0
2019-10	54	0	70	0	0
2019-11	52	0	67	0	0
2019-12	62	0	0	0	0
2020-01	57	29	54	0	0
2020-02	68	0	0	0	0
2020-03	52	0	0	0	0
2020-04	40	33	0	0	0
2020-05	50	25	0	0	0
2020-06	55	0	0	0	0
2020-07	63	0	59	0	0
2020-08	66	34	0	0	0
2020-09	66	51	66	0	0
2020-10	76	40	55	61	0
2020-11	62	51	0	65	0
2020-12	66	43	0	0	0
2021-01	72	30	64	0	0
2021-02	79	49	61	71	0
2021-03	94	57	0	55	0
2021-04	84	39	55	0	0
2021-05	53	55	0	90	0
2021-06	63	42	0	0	0
2021-07	79	40	0	53	0
2021-08	61	39	0	58	0
2021-09	66	32	0	0	0
2021-10	63	0	54	64	0
2021-11	63	39	73	0	0
2021-12	63	50	0	0	0
2022-01	73	44	0	0	0
2022-02	83	47	100	60	0

2022-03	90	57	65	48	0
2022-04	70	50	0	0	0
2022-05	67	70	0	49	0
2022-06	75	47	0	0	0
2022-07	70	0	0	47	0
2022-08	82	46	51	48	0
2022-09	84	45	0	0	0
2022-10	75	44	0	0	0
2022-11	78	46	89	0	0
2022-12	66	38	51	0	0
2023-01	68	55	70	0	0
2023-02	84	42	82	0	0
2023-03	79	55	78	58	0
2023-04	85	63	46	0	0
2023-05	81	77	62	45	0
2023-06	83	77	0	0	0
2023-07	74	55	0	58	0
2023-08	93	74	94	50	0
2023-09	100	66	86	0	0
2023-10	78	68	68	0	0
2023-11	78	50	72	0	0
2023-12	72	41	0	0	0
2024-01	87	55	91	0	0
2024-02	78	64	56	74	0
2024-03	76	45	93	54	0
2024-04	81	87	76	0	0
2024-05	70	51	0	71	0
2024-06	60	59	69	51	0
2024-07	89	43	0	68	0
2024-08	85	59	0	62	100
2024-09	73	89	60	100	0
2024-10	86	100	62	0	0
2024-11	76	89	84	0	0
2024-12	77	71	80	0	0
2025-01	73	63	47	42	0
2025-02	83	0	0	55	0
2025-03	75	61	47	0	88
2025-04	79	88	56	50	100

2025-05	76	67	55	0	0
2025-06	77	79	90	0	0
2025-07	94	72	46	53	0
2025-08	78	100	0	77	0
2025-09	71	53	52	100	0
2025-10	79	53	0	65	0
2025-11	86	77	100	0	0
2025-12	100	71	95	55	0

2.2 Supplementary Table 2

Table S2. Annual relative search interest (RSI) for cardiorenal syndrome.

Year	RSI (United States)	RSI (United Kingdom)	RSI (Canada)	RSI (Australia)	RSI (New Zealand)	ΔRSI (United states) [a]	Cumulative ΔRSI (United States) [b]
2007	4.42	0	0	0	0	-	-
2008	13.33	0	0	0	0	+8.91	+8.91
2009	10.33	0	0	0	0	-3.00	+5.91
2010	37.17	0	0	0	0	+26.84	+32.75
2011	27.92	0	0	0	0	-9.25	+23.50
2012	33.75	0	0	0	0	+5.83	+29.33
2013	44.83	0	0	5.92	0	+11.08	+40.41
2014	46.75	0	0	0	0	+1.92	+42.33
2015	48.42	8.25	0	0	0	+1.67	+44.00
2016	39.83	0	0	0	0	-8.59	+35.41
2017	49.50	2.83	0	0	7.08	+9.67	+45.08
2018	57.50	8.08	6.17	0	0	+8.00	+53.08
2019	64.25	3.33	17.50	5.83	0	+6.75	+59.83
2020	60.08	25.50	19.50	10.50	0	-4.17	+55.66
2021	70.00	39.33	25.58	32.58	0	+9.92	+65.58
2022	76.08	44.50	29.67	21.00	0	+6.08	+71.66
2023	81.25	60.25	54.83	17.58	0	+5.17	+76.83
2024	78.17	67.67	55.92	40.00	8.33	-3.08	+73.75
2025	81.00	65.33	49.00	41.41	15.67	+2.83	+76.58

[a] First-difference (year-over-year change or change from previous year). [b] The running sum of year-over-year changes. n.a., not available.

2.3 Supplementary Table 3

Table S3. Annual mortality rates for ICD-10 113 cause of death code I13.x, I50, and N17-N19 extracted from the CDC WONDER database, 2007-2020.

Year	I13 [a]			I50 [a]			N17-N19 [a]		
	Crude mortality [b]	ΔMortality [c]	Cumulative ΔMortality [d]	Crude mortality [b]	ΔMortality [c]	Cumulative ΔMortality [d]	Crude mortality [b]	ΔMortality [c]	Cumulative ΔMortality [d]
2007	9.9	-	-	920.2	-	-	709.2	-	-
2008	9.4	-0.5	-0.5	925.5	+5.3	+5.3	693.4	-15.8	-15.8
2009	9.4	0	-0.5	895.1	-30.4	-25.1	674.9	-18.5	-34.3
2010	9.1	-0.3	-0.8	904.0	+8.9	-16.2	665.3	-9.6	-43.9
2011	11.7	+2.6	+1.8	912.7	+8.7	-7.5	824.7	+159.4	+115.5
2012	12.5	+0.8	+2.6	910.9	-1.8	-9.3	823.1	-1.6	+113.9
2013	12.7	+0.2	+2.8	949.4	+38.5	+29.2	592.8	-230.3	-116.4
2014	13.8	+1.1	+3.9	969.0	+19.6	+48.8	585.7	-7.1	-123.5
2015	15.5	+1.7	+5.6	1028.6	+59.6	+108.4	597.5	+11.8	-111.7
2016	17.8	+2.3	+7.9	1042.1	+13.5	+121.9	594.5	-3.0	-114.7
2017	22.3	+4.5	+12.4	1081.1	+39.0	+160.9	602.9	+8.4	-106.3
2018	26.0	+3.7	+16.1	1120.1	+39.0	+199.9	609.6	+6.7	-99.6
2019	31.1	+5.1	+21.2	1150.4	+30.3	+230.2	612.4	+2.8	-96.8
2020	37.6	+6.5	+27.7	1262.3	+111.9	+342.1	736.4	+124.0	+27.2

[a] ICD-10 113 mortality cause list: I13, hypertensive heart and chronic kidney disease with heart failure; I50, acute heart failure; N17-N19, renal failure. [b] Per million. [c] First-difference (year-over-year change or change from previous year). [d] The running sum of year-over-year changes from the baseline year (2007) to each subsequent year.

2.4 Supplementary Table 4

Table S4. Annual mortality rates across various ICD-10 113 cause of death code combinations using "AND" logic.

Year	I13&I50 [a]			I13&N17-N19 [a]			I13&I50&N17-N19 [a]		
	Crude mortality [b]	ΔMortality [c]	Cumulative ΔMortality [d]	Crude mortality [b]	ΔMortality [c]	Cumulative ΔMortality [d]	Crude mortality [b]	ΔMortality [c]	Cumulative ΔMortality [d]
2007	4.6	-	-	4.4	-	-	5.2	-	-
2008	4.2	−0.4	−0.4	4.0	−0.4	−0.4	4.7	−0.5	−0.5
2009	4.1	−0.1	−0.5	3.8	−0.2	−0.6	4.7	0.0	−0.5
2010	4.0	−0.1	−0.6	3.7	−0.1	−0.7	4.6	−0.1	−0.6
2011	5.4	+1.4	+0.8	8.5	+4.8	+4.1	8.8	+4.2	+3.6
2012	5.9	+0.5	+1.3	8.6	+0.1	+4.2	8.8	0.0	+3.6
2013	5.9	0.0	+1.3	1.9	−6.7	−2.5	7.4	−1.4	+2.2
2014	6.4	+0.5	+1.8	2.0	+0.1	−2.4	8.0	+0.6	+2.8
2015	7.5	+1.1	+2.9	2.3	+0.3	−2.1	9.4	+1.4	+4.2
2016	8.4	+0.9	+3.8	2.7	+0.4	−1.7	10.6	+1.2	+5.4
2017	10.6	+2.2	+6.0	3.9	+1.2	−0.5	13.5	+2.9	+8.3
2018	12.7	+2.1	+8.1	4.7	+0.8	+0.3	16.2	+2.7	+11.0
2019	14.7	+2.0	+10.1	6.4	+1.7	+2.0	19.6	+3.4	+14.4
2020	18.6	+3.9	+14.0	8.4	+2.0	+4.0	24.9	+5.3	+19.7

[a] Combinations of ICD-10 113 cause of death: I13, hypertensive heart and chronic kidney disease with heart failure; I50, acute heart failure; N17-N19, renal failure. [b] Per million. [c] First-difference (year-over-year change or change from previous year). [d] The running sum of year-over-year changes.

2.5 Supplementary Table 5

Table S5. Annual mortality rates across various ICD-10 113 cause of death code combinations using "OR" logic.

Year	I13/I50 [a]			I13/N17-N19 [a]			I13/I50/N17-N19 [a]		
	Crude mortality [b]	ΔMortality [c]	Cumulative ΔMortality [d]	Crude mortality [b]	ΔMortality [c]	Cumulative ΔMortality [d]	Crude mortality [b]	ΔMortality [c]	Cumulative ΔMortality [d]
2007	930.7	0.0	0.0	719.9	0.0	0.0	1484.7	0.0	0.0
2008	935.7	+5.0	+5.0	703.8	-16.1	-16.1	1482.2	-2.5	-2.5
2009	905.2	-30.5	-25.5	685.2	-18.6	-34.7	1440.4	-41.8	-44.3
2010	914.1	+8.9	-16.6	675.6	-9.6	-44.3	1441.6	+1.2	-43.1
2011	923.1	+9.0	-7.6	832.1	+156.5	+112.2	1562.5	+120.9	+77.8
2012	922.3	-0.8	-8.4	831.9	-0.2	+112.0	1559.6	-2.9	+74.9
2013	962.1	+39.8	+31.4	609.5	-222.4	-110.4	1426.1	-133.5	-58.6
2014	983	+20.9	+52.3	604.1	-5.4	-115.8	1439.1	+13.0	-45.6
2015	1043.9	+60.9	+113.2	618	+13.9	-101.9	1505.9	+66.8	+21.2
2016	1060.5	+16.6	+129.8	618.6	+0.6	-101.3	1517.1	+11.2	+32.4
2017	1103.4	+42.9	+172.7	631.9	+13.3	-88.0	1561.5	+44.4	+76.8
2018	1145.9	+42.5	+215.2	643.3	+11.4	-76.6	1602.5	+41.0	+117.8
2019	1181.3	+35.4	+250.6	651.7	+8.4	-68.2	1635	+32.5	+150.3
2020	1304.9	+123.6	+374.2	789.1	+137.4	+69.2	1867.6	+232.6	+382.9

[a] Combinations of ICD-10 113 cause of death: I13, hypertensive heart and chronic kidney disease with heart failure; I50, acute heart failure; N17-N19, renal failure. [b] Per million. [c] First-difference (year-over-year change or change from previous year). [d] The running sum of year-over-year changes.

2.6 Supplementary Table 6

Table S6. Correlation analysis between relative search interest (RSI) and mortality across various ICD-10 113 cause of death code using "OR" logic.

	ΔRSI and ΔMortality (First-difference analysis) [a]	Cumulative ΔRSI and cumulative ΔMortality [a]	RSI and mortality (Raw series) [a]	RSI and mortality			
				DW-test from OLS residuals [b]	AR(1) coefficient φ [c]	AR(1) modeling [d]	DW-test in AR(1) modeling [e]
**I13**	r=-0.36, 95%CI: -0.76 to 0.24 p=0.23	r=0.80 95% CI: 0.45 to 0.94 p=0.001	r=0.79 95% CI: 0.44 to 0.93 p=0.001	d=0.85 95%CI: 0.00 to 1.90	φ=0.89 95%CI: 0.61 to 1.17 p<0.001	β=1.58 95% CI: -0.47 to 3.63 p=0.11	1.94
**I13/I50**	r=-0.09 95%CI: -0.61 to 0.48 p=0.76	r=0.80 95% CI: 0.45 to 0.94 p=0.001	r=0.78 95% CI: 0.43 to 0.93 p=0.001	d=0.41 95%CI: 0.05 to 0.78	φ=0.38 95%CI: -0.4 to 0.90 p=0.16	β=2.85 95% CI: 0.82 to 4.88 p=0.001	1.94
**I13/N17-N19**	r=-0.47 95%CI: -0.81 to 0.10 p=0.10	r=-0.28 95% CI: -0.72 to 0.32 p=0.35	r=-0.30 95% CI: -0.72 to 0.28 p=0.29	d=0.53 95%CI: 0.06 to 0.99	φ=-0.04 95%CI: -0.60 to 0.53 p=0.89	β=-0.29 95% CI: -2.91 to 2.34 p=0.82	2.08
**I13/I50/N17-N19**	r=-0.41 95%CI: -0.79 to 0.18 p=0.16	r=0.55 95% CI: 0.0002 to 0.85 p=0.051	r=0.53 95% CI: 0.01 to 0.83 p=0.049	d=0.42 95%CI: 0.05 to 0.79	φ=0.32 95%CI: -0.20 to 0.85 P=0.21	β=2.66 95% CI: 0.22 to 5.10 p=0.04	1.97

RSI, relative search interest; DW, Durbin-Watson; OLS, ordinary least squares; AR, autoregressive. [a] Pearson correlation. [b] Across all four ICD-10 definitions, the upper bound of the 95% confidence interval for the DW statistic does not reach 2.0 (ranging from 0.78 to 1.90). This confirms that all series exhibit significant positive autocorrelation, justifying the use of AR(1) modeling for all definitions. [c] The AR(1) coefficient (φ) for I13 is significant at $p < 0.001$, confirming the presence of strong, persistent autocorrelation in the residuals. [d] After accounting for this autocorrelation, the associations between RSI and mortality remain statistically significant across definitions of I13/I50 and I13/I50/N17-N19.[e] The DW statistics improved from below 0.92 in the unadjusted OLS models to between 1.92 and 1.96 in the AR(1) models, indicating successful correction for autocorrelation.

2.7 Supplementary Table 7

Table S7. State-level data: relative search interest (RSI) for cardiorenal syndrome, mortality & age-adjusted mortality, median family income, educational attainment, and insurance coverage.

State	Relative search interest	Mortality per million	Age-adjusted mortality per million	Income [a]	Education [a]	Insurance [a]
Alabama	40	21	19	66,772	26.2	89.5
Alaska	20	4	6	92,648	30.0	87.2
Arizona	40	15	13	73,456	30.3	89.7
Arkansas	40	20	17	62,067	23.8	91.3
California	20	18	17	89,798	34.7	91.9
Colorado	20	16	16	92,752	41.6	91.6
Connecticut	60	10	7	102,061	40.0	94.4
Delaware	60	15	13	84,825	32.7	93.9
District of Columbia	40	44	44	120,337	59.8	96.0
Florida	40	17	11	69,670	30.5	86.7
Georgia	20	16	17	74,127	32.2	86.1
Hawaii	40	15	11	97,813	33.6	96.2
Idaho	40	16	15	70,885	28.7	88.6
Illinois	40	16	14	86,251	35.5	92.5
Indiana	40	18	16	73,265	27.2	92.0
Iowa	40	23	17	79,186	29.3	95.1
Kansas	40	13	11	77,620	33.9	90.8
Kentucky	40	14	12	65,893	25.0	94.3
Louisiana	40	23	21	65,427	24.9	90.2
Maine	20	12	9	76,192	32.5	92.1
Maryland	40	16	15	105,790	40.9	93.6
Massachusetts	60	10	7	106,526	44.5	97.1
Michigan	60	21	17	75,470	30.0	94.4

Minnesota	60	16	13	92,692	36.8	95.3
Mississippi	40	31	29	58,923	22.8	87.0
Missouri	60	16	13	72,834	29.9	90.3
Montana	20	8	7	72,773	33.1	91.4
Nebraska	60	17	13	80,125	32.5	91.5
Nevada	20	14	14	74,077	25.5	88.5
New Hampshire	40	9	7	97,001	37.6	93.6
New Jersey	40	10	8	104,804	40.7	91.7
New Mexico	40	10	9	62,611	28.1	90.1
New York	40	16	13	87,270	37.5	93.9
North Carolina	40	15	13	70,978	32.0	88.3
North Dakota	40	18	14	86,798	30.7	93.3
Ohio	60	21	17	74,391	28.9	93.8
Oklahoma	20	57	51	67,511	26.1	84.7
Oregon	40	18	14	80,630	34.4	92.9
Pennsylvania	60	15	10	80,996	32.3	94.5
Rhode Island	40	16	12	89,330	35.0	95.6
South Carolina	40	21	19	68,813	29.0	88.7
South Dakota	40	26	20	77,042	29.3	90.5
Tennessee	60	30	27	68,793	28.2	89.3
Texas	20	16	18	76,073	30.7	81.8
Utah	20	7	9	84,590	34.7	90.5
Vermont	60	24	18	83,023	39.7	95.5
Virginia	40	13	13	93,284	39.5	91.3
Washington	40	22	20	92,422	36.7	93.2
West Virginia	100	15	11	61,707	21.3	93.5
Wisconsin	60	18	14	80,844	30.8	94.6
Wyoming	20	8	7	81,290	28.2	88.8

[a] Median family income (in U.S. dollars). educational attainment (percentage of the population aged ≥25 years with bachelor's degree or higher), and health insurance coverage rate (percentage of the population with any form of insurance). All data are for the year 2020 and were obtained from the U.S. Census Bureau (https://data.census.gov/table).
